# Probability Matching as a Computational Strategy Used in Perception

**DOI:** 10.1371/journal.pcbi.1000871

**Published:** 2010-08-05

**Authors:** David R. Wozny, Ulrik R. Beierholm, Ladan Shams

**Affiliations:** 1 Max Planck Institute for Human Cognitive and Brain Sciences, Leipzig, Germany; 2 Biomedical Engineering IDP, University of California Los Angeles, Los Angeles, California, United States of America; 3 Gatsby Computational Neuroscience Unit, University College London, London, United Kingdom; 4 Department of Psychology, University of California Los Angeles, Los Angeles, California, United States of America; New York University, United States of America

## Abstract

The question of which strategy is employed in human decision making has been studied extensively in the context of cognitive tasks; however, this question has not been investigated systematically in the context of perceptual tasks. The goal of this study was to gain insight into the decision-making strategy used by human observers in a low-level perceptual task. Data from more than 100 individuals who participated in an auditory-visual spatial localization task was evaluated to examine which of three plausible strategies could account for each observer's behavior the best. This task is very suitable for exploring this question because it involves an implicit inference about whether the auditory and visual stimuli were caused by the same object or independent objects, and provides different strategies of how using the inference about causes can lead to distinctly different spatial estimates and response patterns. For example, employing the commonly used cost function of minimizing the mean squared error of spatial estimates would result in a weighted averaging of estimates corresponding to different causal structures. A strategy that would minimize the error in the inferred causal structure would result in the selection of the most likely causal structure and sticking with it in the subsequent inference of location—“model selection.” A third strategy is one that selects a causal structure in proportion to its probability, thus attempting to match the probability of the inferred causal structure. This type of probability matching strategy has been reported to be used by participants predominantly in cognitive tasks. Comparing these three strategies, the behavior of the vast majority of observers in this perceptual task was most consistent with probability matching. While this appears to be a suboptimal strategy and hence a surprising choice for the perceptual system to adopt, we discuss potential advantages of such a strategy for perception.

## Author Summary

For any task, the utility function specifies the goal to be achieved. For example, in taking a multiple-choice test, the utility is the total *number* of correct answers. An optimal decision strategy for a task is one that maximizes the utility. Because the utility functions and decision strategies used in perception have not been empirically investigated, it remains unclear what decision-making strategy is used, and whether the choice of strategy is uniform across individuals and tasks. In this study, we computationally characterize a decision-making strategy for each individual participant in an auditory-visual spatial localization task, where participants need to make implicit inferences about whether or not the auditory and visual stimuli were caused by the same or independent objects. Our results suggest that a) there is variability across individuals in decision strategy, and b) the majority of participants appear to adopt a probability matching strategy that chooses a value according to the inferred probability of that value. These results are surprising, because perception is believed to be highly optimized by evolution, and the probability matching strategy is considered “suboptimal” under the commonly assumed utility functions. However, we note that this strategy is preferred (or may be even optimal) under utility functions that value learning.

## Introduction

Human performance in perceptual tasks is often benchmarked by optimal strategies. An optimal strategy is one that performs best with respect to its objectives or maximizes expected reward or equivalently, minimizes a cost function [1], [2]. Previous studies have investigated whether performance in perceptual tasks is consistent with normative models that use maximum likelihood estimation (MLE) [3]–[5], Bayesian inference [6]–[8], signal detection theory [9]–[11], or other computational frameworks. These previous studies either implicitly or explicitly assume a certain cost/utility function that defines the optimal decision. In contrast, the question of which utility/cost function is used by the nervous system for perceptual tasks has not been systematically investigated [but see 12], [13].

In this study we aim to computationally characterize human perceptual decision making strategies. As different strategies may be used across individuals, we characterize the strategy used by each individual observer instead of modeling the behavior of an “average observer”. We used a spatial localization task, as it is simple and fundamental to perceptual processing. While spatial localization has been studied extensively, it has not been investigated in the context of decision making strategies. In nature, at any given moment, we are typically exposed to both visual and auditory stimuli, and scene perception and analysis requires simultaneous inference about the location of auditory and visual stimuli (as well as other sensory stimuli such as tactile, and olfactory). Therefore, multisensory spatial localization represents a task that the perceptual system is implicitly engaged in at all times. This task is particularly useful for probing decision-making strategies because it involves an automatic causal inference about the sources of stimuli, and distinct patterns of behavior corresponding to different strategies. For each observer we examined which of three plausible decision making strategies best accounts for their performance. We use a Bayesian causal inference model of multisensory perception [14] to quantify subjects' responses as one of three strategies as well as compare them to qualitative predictions of such strategies.

**Figure 1:**
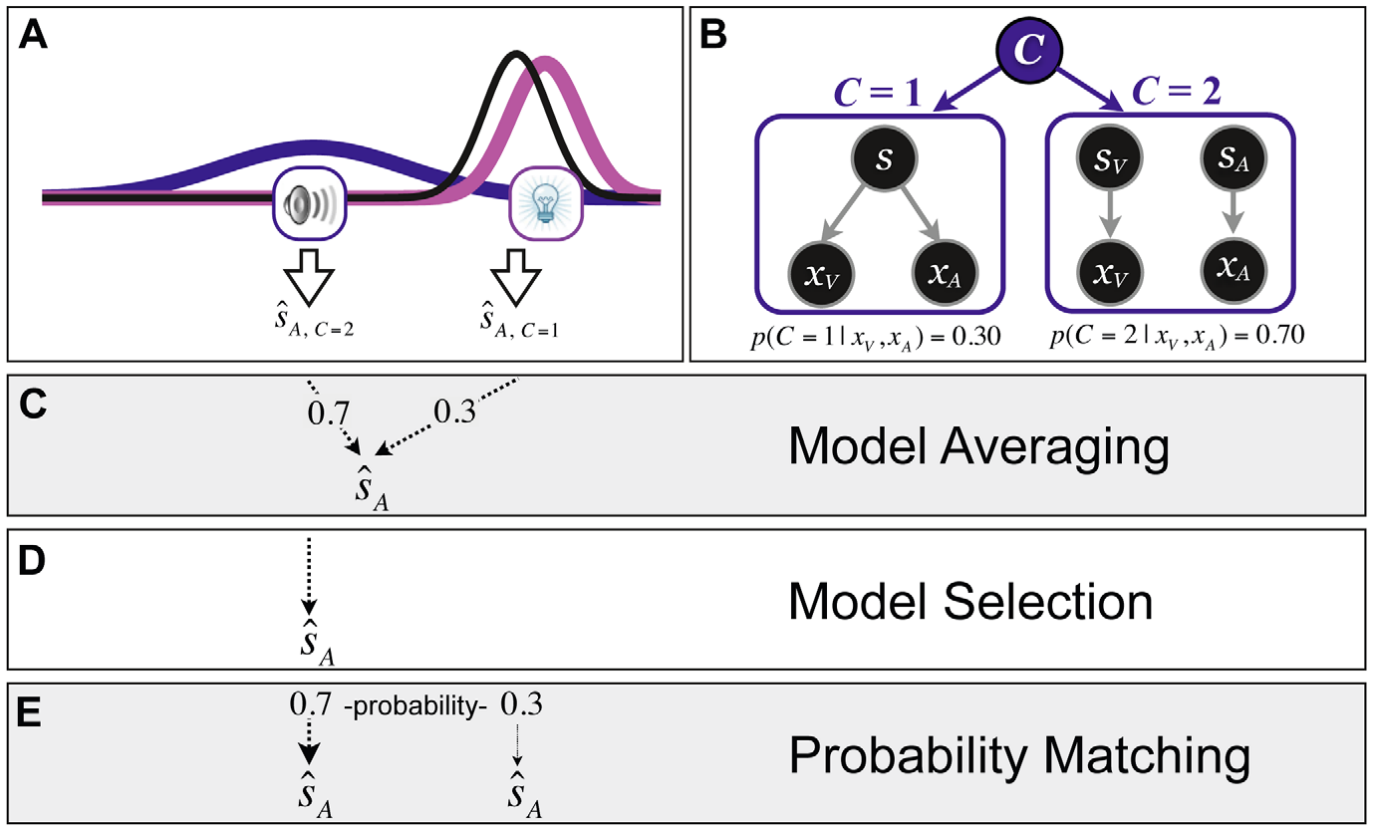
Illustration of the three different decision strategies for producing an auditory estimate of location. (A) A schematic example of sensory representations on a trial with a certain discrepancy between the auditory and visual stimuli. The lightbulb and speaker symbols represent the visual and auditory stimulus locations, respectively. The visual and auditory likelihoods are shown in magenta and blue, respectively. For the sake of simplicity, here we assume that the prior distribution is non-informative (uniform). Therefore, in the case of a common cause (*C* = 1), i.e., when the two sensory signals are fused to obtain an estimate, a single Gaussian posterior distribution is obtained which is shown in black. The estimate of the location of sound, 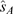 is the mean of the black distribution. In contrast, in the independent cause scenario (*C* = 2), this estimate is the mean of the blue distribution. (B) The generative model for the causal inference model. *C* = 1: One cause can be responsible for both visual and auditory signals, *x_V_* and *x_A_*. *C* = 2: Alternatively, two independent causes may generate the visual and auditory cues. The probability of each causal structure can be computed using Bayes' Rule (see Eq. 1). Hypothetical posterior probabilities for the stimuli in (A) are given at the bottom of each causal structure. For model averaging (C), the final auditory estimate would be a weighted average of the two estimates, with each estimate weighted by the probability of its causal structure. For model selection (D), an estimate is derived based on the most probable model, in this case the independent model (*C* = 2). For probability matching (E), the final auditory estimate in this example would be equal to the independent model estimate (*C* = 2) 70% of the time, and equal to the common cause model estimate (*C* = 1) 30% of the time. Visual estimates are produced likewise.

One strategy tested was the objective of minimizing the mean squared error. This is a commonly used loss function in normative models of human behavior [3], [4], [7], [15], [16]. It assumes that the nervous system tries to minimize the squared error on average. This utility function in the context of our task implies *model averaging*, i.e., weighted averaging of the estimate derived from two different causal structures [14]: a common cause hypothesis and an independent causal hypothesis, each weighted by their respective probability (see Figure 1c).

Another strategy we tested was to minimize the error in the inferred causal structure as well as the error in the spatial estimate. This strategy in the context of our task translates into *model selection* [17], [18]. This strategy also maximizes the consistency in the inference process [13]. In our task, model selection maximizes consistency between the causal structure chosen and the estimate of location. In this strategy, the estimate of location is purely based on the causal structure that is deemed to be most likely (see Figure 1d).

The third strategy tested is *probability matching* [19]–[22]. This strategy has been reported to be used by humans in a variety of cognitive tasks. In these tasks, probability matching refers to the phenomenon in which observer's probability of a given response matches the probability of appearance of the given target. For example, if the task is to predict which one of two colored lights will be presented in each trial, in an experiment in which each color is presented with a certain probability, then the participant's frequency of predicting each color will be consistent with the relative frequency of the presentation of the color. For a situation where a green light is presented 70% of the time, and a blue light 30% of the time, probability matching behavior would predict the green light on approximately 70% of trials. This strategy is considered to be sub-optimal in terms of economic and utility theory because once it is known that the green light is presented more often, observers should predict the green light on all trials to maximize their utility or gain (.70 proportion correct vs. .70×.70+.30×.30 = .58 proportion correct). Therefore, probability matching has not received much attention in the study of perception—which is generally considered to be highly optimized by evolution [but see 23]–[25 for evidence in visual selective attention]. Nonetheless, because of its implication in the decision making literature, we included this strategy in our analysis. In our task, this strategy would translate into choosing a causal structure according to the probability of the underlying causal structure. Thus, this strategy is one step removed from matching the probability of response outcomes but rather matches the probability of the implicit causal structure (see Figure 1e).

## Methods

### Ethics Statement

This study was conducted according to the principles expressed in the Declaration of Helsinki. All participants in the experiment provided written informed consent in approval with the UCLA Institutional Review Board.

### Participants, Procedure and Stimuli

One hundred and forty six subjects participated in the experiment. We used a large sample because we wanted to be able to detect even small subpopulations (e.g., a small percentage of observers) who may adopt a different strategy. Participants sat at a desk in a dimly lit room with their chins positioned on a chin-rest 52 cm from a projection screen. The screen was a black acoustically transparent cloth subtending much of the visual field (134° width×60° height). Behind the screen were 5 free-field speakers (5×8 cm, extended range paper cone), positioned along azimuth 6.5° apart, 7° below fixation. The middle speaker was positioned below the fixation point, and two speakers were position to the right and two to the left of the fixation. The visual stimuli were presented overhead from a ceiling mounted projector set to a resolution of 1280×1024 pixels with a refresh rate of 75 Hz.

The visual stimulus was a white-noise disk (.41 cd/m^2^) with a Gaussian envelope of 1.5° FWHM, presented 7° below the fixation point on a black background (.07 cd/m^2^), for 35 ms. The visual stimuli were always presented so that their location overlapped the center of one of the five speakers behind the screen positioned at −13°, −6.5°, 0°, 6.5° 13°. Auditory stimuli were ramped white noise bursts of 35 ms measuring 69 dB(A) sound pressure level at a distance of 52 cm. The speaker locations were unknown to the participants.

In order to familiarize participants with the task, each session started with a practice period of 10 randomly interleaved trials in which only an auditory stimulus was presented at a variable location, and subjects were asked to report the location of the auditory stimulus.

Practice was followed by 525 test trials that took about 45 minutes to complete. 15 repetitions of 35 stimulus conditions were presented in pseudorandom order. The stimulus conditions included 5 unisensory auditory locations, 5 unisensory visual locations, and all 25 combinations of auditory and visual locations (bisensory conditions). On bisensory trials, subjects were asked to report *both* the location of auditory stimulus and the location of visual stimulus in sequential order. The order of these two responses was consistent throughout the session, and was counter-balanced across subjects. Subjects were told that “the sound and light could come from the same location, or they could come from different locations.” As a reminder, a blue ‘S’ or green ‘L’ was placed inside the cursor to remind subjects to respond to the sound or light respectively.

Each trial started with fixation cross, followed after 750–1100 ms by the presentation of the stimuli. After 450 ms, fixation was removed and a cursor appeared on the screen vertically just above the horizontal line where the stimuli were presented and at a random horizontal location in order to minimize response bias. The cursor was controlled by a trackball mouse placed in front of the subject, and could only be moved in the horizontal direction. Participants were instructed to “move the cursor as quickly and accurately as possible to the exact location of the stimulus and click the mouse”. This enabled the capture of continuous responses with a resolution of 0.1 degree/pixel.

### Causal Inference Model

We used a Bayesian causal inference model of multisensory perception augmented with one of the three decision strategies described above to classify the decision making strategy used by each participant. Details of the model can be found elsewhere [14], but in summary, each stimulus or event, 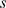, in the world causes an underlying noisy sensory estimate, 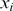, of the event (where *i* is indexed over sensory channels). Similar to [14], the sensory estimate for our task is the perceived location of the auditory and visual stimuli. We use a generative model to simulate experimental trials and subject responses by performing 10,000 Monte Carlo simulations per condition. Each individual sensation is modeled using the likelihood function 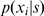. Trial-to-trial variability is introduced by sampling the likelihood from a normal distribution around the true sensory location, analogous to having auditory and visual sensory channels corrupted by independent Gaussian noise with parameters σ_A_ and σ_V_ respectively. We assume there is a prior bias for the central location, as modeled by a Gaussian distribution centered at 0°. The strength of this bias, σ_P_, is a free parameter. The causal inference model infers the underlying causal structure, *C*, of the environment based on the available sensory evidence and prior knowledge using Bayes' rule shown in Equation 1.
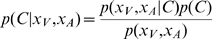
(1)
Figure 1 shows a schematic example for a bimodal stimulus presentation. The competing causal structures are shown in Figure 1-B, where either the sensations could arise from a common cause (*C* = 1, Figure 1-B left), or from independent causes (*C* = 2, Figure 1-B right). The optimal estimates for the visual and auditory locations are given in Equation 2 for the common cause model, and Equation 3 for the independent model.
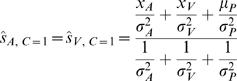
(2)

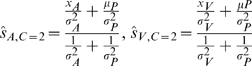
(3)The difference in our modeling compared to [14] is in producing the final spatial location estimates. The goal of the nervous system is to come up with the best estimates of stimulus locations, 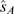 and 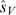. If the objective is to minimize mean squared error of the spatial estimates, then the optimal estimates are obtained by model averaging:
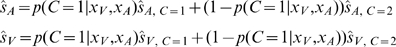
(4)where 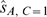 is the optimal estimate of auditory location given there is a single cause (Eq. 2), and 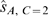 is the optimal estimate of auditory location given there are independent causes (Eq. 3) (see Figure 1-A). The final estimate 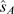 is a weighted average of the two estimates each weighted by the posterior probability of the respective causal structure (Figure 1-C). 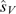 is computed likewise.

In model selection strategy (Figure 1-D), on each trial, the location estimate is based purely on the causal structure that is more probable given the sensory evidence and prior bias about the two causal structures:
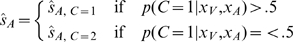
(5)For probability matching (Figure 1-E), location estimates are based on selecting a causal structure based on the inferred posterior probability of the structure. In other words, the selection criterion is stochastic and no longer deterministic. This can be achieved by using a variable selection criteria, 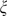, that is sampled from a uniform distribution on each trial.
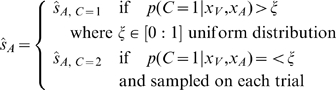
(6)All three models described above have four free parameters: the standard deviation of the auditory and visual likelihoods σ_A_ and σ_V_, the standard deviation of the prior over space, σ_P_, and the prior probability of a common cause, p(C = 1) = *pcommon*. We fit subject responses to the causal inference model for each of the three strategies and determine the best strategy based on the maximum likelihood fit for each subject (see Supplementary Text S1 for a detailed description of the fitting procedure).

**Figure 2:**
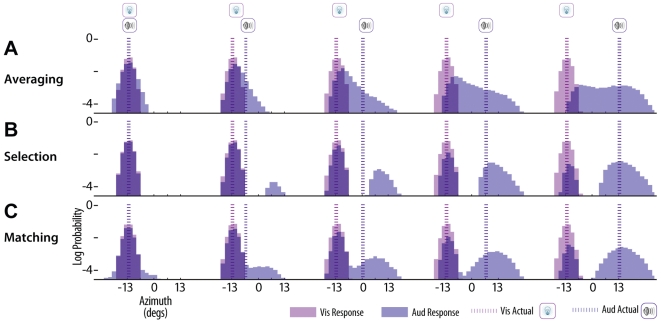
Simulated response patterns. Simulated response distributions for the three strategies: model averaging (A), model selection (B), and probability matching (C). Distributions are created from 10,000 samples per condition, using mean subject parameters [σ_V_ = 2.5° σ_A_ = 10.1° σ_P_ = 33.0° *pcommon* = 0.57], and only changing the decision strategy. Five bimodal conditions are shown for each strategy with the visual stimulus to the far left, and the auditory stimulus growing in discrepancy from the left to the right columns. Vertical blue and magenta dotted lines along with the speaker and lightbulb icons show the true location of the auditory and visual locations, respectively. The predicted log-probability of response is shown by the shaded bars for both the visual (magenta) and auditory (blue) response distributions, with overlaps shown in a darker shade of blue.

The three decision strategies produce distinct patterns of responses across trials and stimulus conditions. Figure 2 shows response distributions for each of the three strategies generated by Monte Carlo simulations for a few stimulus conditions. For these simulations, we used parameter values that are typically found when fitting human observers data. Because vision has a much higher precision in this task than hearing, visual estimates are not affected much by sound. Therefore, we focus our attention on the auditory responses shown in blue. In general, the model averaging strategy mostly has unimodal response distributions, and in conditions with moderate conflict between the visual and auditory stimuli, the auditory responses are shifted in the direction of the visual stimulus (Figure 2-A). In contrast, for the model selection strategy, the auditory responses are mostly bimodal and consistent with either unisensory auditory responses, or complete fusion of the stimuli (Figure 2-B). The probability mass around each peak varies consistently with the expected probability of each causal structure. In other words, for conditions in which the discrepancy between visual and auditory locations is large, and thus the probability of a common cause is low, there is a large probability mass at the auditory location, and in conditions where the conflict is small, and thus the probability of common cause is high, there is a much larger probability mass around the visual capture location (i.e., location shifted towards visual stimulus). The fixed selection criterion results in distinct separation between the two auditory response distribution modes. For any probability of a common cause greater 0.5, the auditory response will be fused with the visual response. Similarly, the probability matching strategy also shows bimodal auditory response distributions (Figure 2-C). However, in contrast with model selection, the modes are not as distinct. Due to the variable model selection criteria (*ξ*), even when the probability of a common cause is high, there is a small probability of providing an auditory response consistent with the independent causal structure. Due to the high uncertainty in the auditory signals (i.e., large variance of auditory likelihood), this can even be observed when the stimulus locations are identical (left column).

## Results

For each participant, each of the decision strategy models was fitted to the data, and the observer was classified by the strategy that explained the data best. In order to be highly confident in the classifications, for an observer to be included in the sample we required that the log-likelihood difference between the best-fitting model and the second best-fitting model exceed a value of 3—which is considered substantial evidence for the support of one model vs. another [26]. In Table 1, we report the results from 110 participants whose data met this criterion. Among these participants, on average the log-likelihood difference between the top two best-fitting models was 24.6 (median 17.6), which is in the range considered as decisive evidence for a model relative to another model [26]. On average, the best fitting model accounted for 83% of the variance in the individual participant's data (generalized coefficient of determination [27]: R^2^ = 0.83±0.11). The model fits for the probability-matching group data is also shown for all stimulus conditions in Supplementary Figure S1. Therefore, the best-fitting model fitted the data very well, and the classifications were highly reliable.

**Table 1:** Summary of participant strategy classification.

	All subjects	Females	Males	Age (μ±SD)
Matching	82 (75%)	57 (75%)	25 (74%)	20.9±3.0
Selection	10 (9%)	7 (9%)	3 (9%)	20.4±2.1
Averaging	18 (16%)	12 (16%)	6 (17%)	21.5±3.3
Total	110	76	34	20.9±3.0

The number of participants classified as utilizing the matching, selection, or averaging strategy is provided in Table 1. Probability matching is the decision strategy used by the vast majority of observers (82/110). Model averaging was second followed by model selection. The proportion of males and females is not significantly different for each strategy (two-sample test for equality of proportions, p>0.05). The difference in distribution of ages among the three groups was also statistically insignificant (two-sample Kolmogorov-Smirnov test, p>0.05). It should be pointed out that these results are not sensitive to the subject exclusion criterion described above. The results remain qualitatively the same even if we do not exclude any participants at all: N = 146, matching = 64%; selection = 18%; averaging = 18%, or if we use other exclusion criteria (e.g., margin of 10 instead of 3: N = 82, matching = 79%; selection = 5%; averaging = 16%).

We also tested whether the model selection strategy could explain the data better than the other two strategies if we allow a bias in choosing a model (i.e., if the criterion can take on any value as a free parameter, rather than fixed at .5 as in Equation 5). Despite the additional free parameter for this model, we find similar proportions of categorization: N = 110, matching = 72%; selection = 13%; averaging = 15% – and after applying Bayesian Information Criteria regularization for the additional free parameter: matching = 72%; selection = 11%; averaging = 17%.

## Discussion

We aimed to gain insight into the decision making strategy used in a perceptual task, by comparing three strategies and testing which one accounts best for the observers' data. Our computational modeling tools allow us to perform this type of analysis for each individual observer. Perceptual functions, in particular the basic ones that are shared across species (and arguably key to the survival of the organism) such as spatial localization, are often thought to be optimal. Perceptual functions are evolutionarily old and thus, it is argued that there has been sufficient amount of time for them to have been optimized by evolution [28], and indeed several studies have shown a variety of perceptual tasks to be “statistically optimal.” For the same reason, it is also expected that the optimized and automated perceptual processes to be largely uniform across individuals.

We examined the decision strategies in an auditory-visual spatial localization task on a large sample of observers consisting of 110 individuals. First, we found that not all observers appear to utilize the same strategy. This variability across individuals suggests that this localization process is not predestined or hard-wired in the nervous system. More importantly, the vast majority of participants seem to use a probability matching strategy. This finding is surprising because this strategy is not statistically optimal in the conventional sense.

Why should the majority of individuals use a “suboptimal” strategy in this basic task? To address this question, it is best to step back and re-examine the notion of optimality. While a probability matching strategy may not be optimal in a static environment, it may be optimal or close to optimal in a dynamic one [29], and especially useful in exploring patterns in the environment. Humans instinctively have the tendency to search for regularities in random patterns [30]–[33], and it has been suggested that probability matching results from the addition of an “informatic” utility that considers learning and exploring an important component in survival and ecological rationality [34]. Thus, while probability-matchers might look irrational in the absence of predictable patterns, they would have a higher chance of finding patterns if they exist [19]. In the context of our experiment, although the stimuli were uniformly random, perhaps the matchers subconsciously explore for patterns within the stimuli.

The observed probability matching behavior suggests that the nervous system samples from a distribution over model hypotheses on each trial. Sampling-based representational coding has been proposed to account for neurophysiological phenomena such as spontaneous neural activity [35] and variability in neural responses [36], as well as other stochastic perceptual phenomena such as bistability [37], [38]. Alternatively, it is conceivable that a case-based selection rule [39] that, on each trial, chooses the most appropriate model from an earlier experience (not necessarily from the current experiment) resembling the current sensations, would produce this behavior.

While probability matching was the modal response strategy found in the current study, we are not claiming that probability matching is used in all perceptual tasks, or even in all spatial tasks. Optimal performance in perceptual tasks has been reported by some previous studies. A recent study found observers' behavior to be consistent with the expected loss function in a visual discrimination task [40], however, the results are ambiguous with respect to the specific decision making strategy utilized (averaging, selection, and probability matching) as they would make similar predictions. Knill, in a study of perception of slant from compression and binocular disparity cues [41], reported optimal performance. In this study, which used an almost identical structure inference model to the one used here, observers' responses were explained well by model averaging. However, probability matching was not considered, and regarding model selection vs. averaging, the author points out that determining exactly which strategy was used by the participants is difficult. Perhaps most relevant to our current findings is a previous study of auditory-visual spatial localization in which the observers' performance was found to be consistent with model averaging [14]. Although model selection and probability matching were not tested, the response profiles were unimodal and thus, inconsistent with these strategies. The sample size was relatively small in this study (N = 19), yet together with the aforementioned studies, these findings raise the question of what are the factors that influence the decision-making strategy adopted by observers. It is likely that the specific strategy used by participants depends on the context, instruction, prior experience, and many other factors [42]. Landy et al. [43] found that stimulus variability and unpredictability from trial to trial can result in adoption of a variety of suboptimal strategies by participants in a texture orientation perception task. Even for a given context, stimuli, and instruction (as in this experiment), some subjects' construal of the task may affect their utility/cost function. The specific computational constraints such as criteria of speed and accuracy could also favor the use of one strategy over another. Also in our study, subjects had to make sequential reports of both modalities requiring responses to be held in working memory, which has been suggested to have a role in the decision process [19], [32], [44]. The specific factors influencing perceptual decision making strategies is an open question for future studies.

Probability matching has been shown to happen when people's response probability matches the relative frequency of the presented stimuli. Here we show that the nervous system can even match the probability of a more abstract construct such as the probability of causal structure of the stimuli which is one step removed from the stimuli themselves. This finding suggests that probability matching may be a general decision-making strategy operating at multiple levels of processing. The results of this study altogether suggest that the nervous system does not necessarily use the commonly assumed least squared error cost function in perceptual tasks, and underscore the importance of considering alterative objectives when evaluating perceptual performance.

## Supporting Information

Text S1 Model fitting and goodness of fit procedure.
(0.03 MB DOC)

Figure S1 Model fits to probability matching group. Shaded areas show the log-probability of response for the 82 subjects classified as using a probability matching strategy. Thick lines show the model fits averaged across individual subject fits. Vertical blue and magenta dotted lines show the location of the auditory and visual stimulus, respectively. The first row shows the five unimodal auditory conditions, ordered from leftmost to rightmost positions along the azimuth as shown by the blue vertical dotted line. The first column shows the five unimodal visual conditions, ordered from leftmost (top) to rightmost (bottom) as shown by the magenta vertical dotted line. The central 25 plots show data from the bisensory conditions with both the visual (magenta) and auditory (blue) response distributions.
(2.70 MB TIF)
