# Supplementary material for: Probability Matching as a Computational Strategy Used in Perception
Source: PLoS Comput Biol. 2010 Aug 5;6(8):e1000871. doi: 10.1371/journal.pcbi.1000871 (PMC2916852; doi:10.1371/journal.pcbi.1000871)
Supplement: Text S1 — Model fitting and goodness of fit procedure. (0.03 MB DOC) [file pcbi.1000871.s001.doc]

**SUPPLIMENTARY MATERIAL**

*Model fitting procedure*

For each subject, 4 free parameters were fitted to the data: the standard deviation of the auditory and visual likelihoods σ_A_ and σ_V_, the standard deviation of the prior over space, σ_P_, and the prior probability of a common cause, p(C=1) = *pcommon*. The parameters were optimized using a simplex minimization search in Matlab over the likelihood of the model. Monte-Carlo simulations were used to calculate the likelihood of the model given the data:

[S1]

For each step of the minimization algorithm, 10,000 trials per experimental condition were simulated from the generative model, and inference was performed according to the Bayesian inference model, generating 10,000 model response simulations per condition. Simulated auditory and visual responses were binned in 1 degree intervals, and the normalized histograms were fitted with cubic splines to produce likelihood functions given the current model and parameters. This provides 60 likelihood functions (5x5x2+5+5, one for each response condition) similar to the thick solid lines of the model predictions shown in supplementary Figure S1. The sum of the negative log-likelihood for the observed responses (900 data-points per subject) was minimized as the objective function for the parameter fits. This procedure was done separately for each of the three decision strategies: model averaging, model selection, and probability matching models. Subjects were classified under the decision strategy that had the overall best fit.

*Goodness of fit*

The generalized coefficient of determination, R^2^, as described in [27] was used to quantify the goodness of fit for the best-fitting model. This provides a continuous measure ranging from zero (corresponding to worst fit) to one (corresponding to best fit) based on the maximum likelihood criteria of fit, without binning the data. The formula used is shown in Equation S2:

[S2]

where is the number of data-points, and and denote the log-likelihoods of the fitted and the null model, respectively. The mean of data was used as the null model (this model explains none of the variance in the data). The generalized R^2^ is interpreted as the proportion of variance in the data that is explained by the model.
