# Supplementary figures and images for: Probability Matching as a Computational Strategy Used in Perception
Source: PLoS Comput Biol. 2010 Aug 5;6(8):e1000871. doi: 10.1371/journal.pcbi.1000871 (PMC2916852; doi:10.1371/journal.pcbi.1000871)

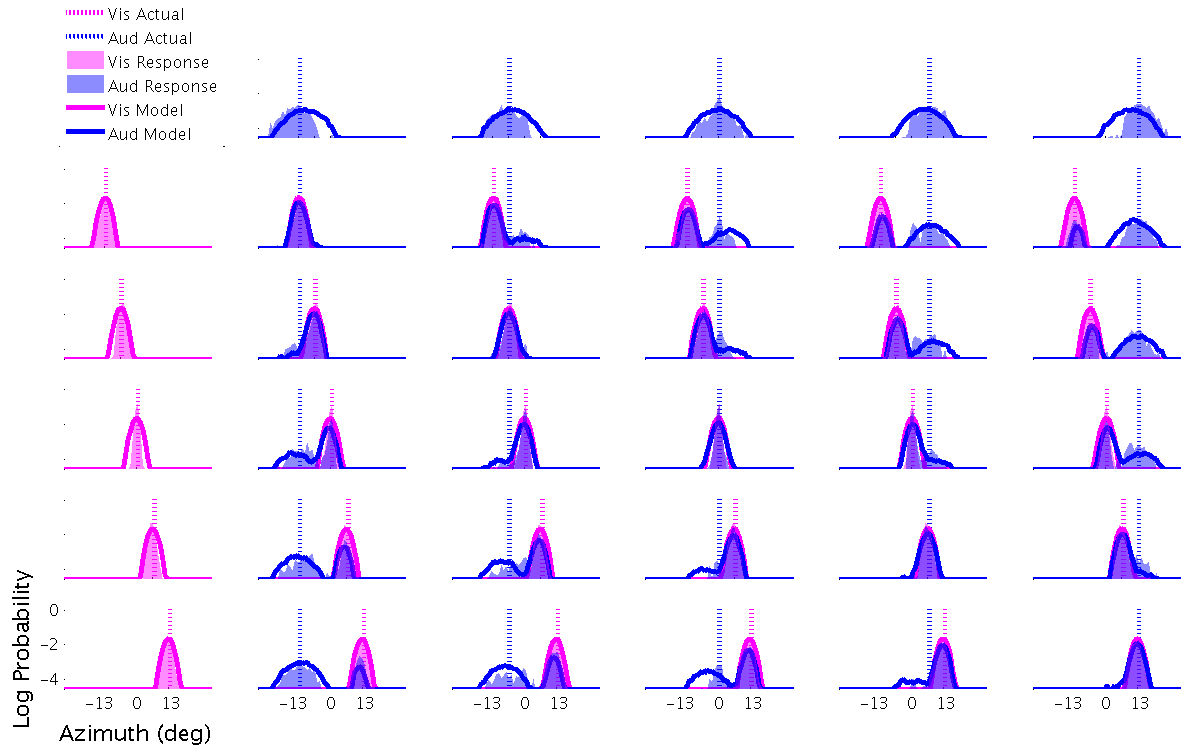

Supplement: Figure S1 — Model fits to probability matching group. Shaded areas show the log-probability of response for the 82 subjects classified as using a probability matching strategy. Thick lines show the model fits averaged across individual subject fits. Vertical blue and magenta dotted lines show the location of the auditory and visual stimulus, respectively. The first row shows the five unimodal auditory conditions, ordered from leftmost to rightmost positions along the azimuth as shown by the blue vertical dotted line. The first column shows the five unimodal visual conditions, ordered from leftmost (top) to rightmost (bottom) as shown by the magenta vertical dotted line. The central 25 plots show data from the bisensory conditions with both the visual (magenta) and auditory (blue) response distributions. (2.70 MB TIF) [file pcbi.1000871.s002.tif]
